# Supplementary material for: Mapping the evolution of fertility support policies in China: A content and instrumental analysis
Source: PLoS One. 2025 Oct 9;20(10):e0332137. doi: 10.1371/journal.pone.0332137 (PMC12510515; doi:10.1371/journal.pone.0332137)
Supplement: S1 Appendix — (ZIP) [file pone.0332137.s001.zip › S1 Appendix. 226 original policy documents/3-国家人口和计划生育委员会办公厅关于转发《吉林省人口计生委关于城乡协调推进人口和计划生育“三结合”开展“五关爱”活动情况的报告》的通知(FBM-CLI-4-66165).doc]

国家人口和计划生育委员会办公厅关于转发《吉林省人口计生委关于城乡协调推进人口和计划生育“三结合”开展“五关爱”活动情况的报告》的通知

**发布部门：** [国家人口和计划生育委员会(含国家计划生育委员会)(已撤销)](https://www.pkulaw.com/law/chl?Aggs.IssueDepartment=60304) [机构沿革](javascript:void(0))

**发布日期：**2005.11.03

**实施日期：**2005.11.03

**时效性：** [现行有效](https://www.pkulaw.com/law/chl?Aggs.TimelinessDic=01)

**效力级别：** [部门工作文件](https://www.pkulaw.com/law/chl?Aggs.EffectivenessDic=XE0303)

**法规类别：** [计划生育管理](https://www.pkulaw.com/law/chl?Aggs.Category=09705)

国家人口和计划生育委员会办公厅关于转发
《吉林省人口计生委关于城乡协调推进人口和计划生育
“三结合”开展“五关爱”活动情况的报告》的通知

各省、自治区、直辖市人口计生委，计划单列市、新疆生产建设兵团人口计生委，解放军、武警部队人口和计划生育领导小组办公室，中直机关、中央国家机关人口计生委：
　　20世纪90年代，吉林省在人口和计划生育工作中总结出“三结合”的经验在全国推广后，对提高人口和计划生育工作的整体水平，促进工作思路和工作方法的“两个转变”，起到了积极的作用。2004年初，吉林省针对新形势新任务，提出城乡协调推进“三结合”开展“五关爱”活动，是贯彻落实“三个代表”重要思想和科学发展观的重要举措；是人口和计划生育工作实现好、维护好、发展好人民群众根本利益的具体体现；是人口和计划生育工作新机制建设的有效载体。吉林省在推进“三结合”开展“五关爱”活动中，坚持政府领导、部门配合、项目运作、科学管理、典型引路、资金保障、全面推进的运作方式，值得各地借鉴。
　　现将吉林省人口计生委《关于城乡协调推进人口和计划生育“三结合”开展“五关爱”活动情况的报告》转发给你们，供参考。
　　附件：《吉林省人口计生委关于城乡协调推进人口和计划生育“三结合”开展“五关爱”活动情况的报告》
　　　　　　　　　　　　　　　　　　　　　　　　　　人口计生委办公厅
　　　　　　　　　　　　　　　　　　　　　　　　　　二00五年十一月三日
　　附件：

关于城乡协调推进人口和计划生育“三结合”

开展“五关爱”活动情况的报告

（吉林省人口计生委）

　　几年来，在省委、省政府的领导下，在国家人口计生委的指导下，我省总结推广了城乡协调推进人口和计划生育“三结合”、“五关爱”活动的经验和做法，推动了人口计生工作新机制的建设，促进了低生育水平的稳定和人口问题的统筹解决，取得了明显成效。
　　最近，省委、省政府下发了《关于在全省城乡深入开展人口和计划生育“三结合”、“五关爱”活动的意见》，对这项活动提出了明确要求。
　　现将我省城乡协调推进人口和计划生育“三结合”开展“五关爱”活动的情况报告如下：
　　一、城乡协调推进“三结合”开展“五关爱”活动的具体内容
　　多年来，我省人口出生率、自然增长率和妇女总和生育率一直稳定在较低水平，但在人口素质、人口结构、人口分布等方面还存在着不容忽视的矛盾和问题。根据基层对育龄群众的问卷调查结果分析，群众的法律意识和婚育观念发生了深刻变化，更加关心优生优育、生殖健康和生活质量。
　　面对新形势，2003年底，我省在整合以往实践经验的基础上，提出城乡协调推进“三结合”开展“五关爱”活动，这项活动把满足育龄群众对计划生育/生殖健康的需求同解决当前人口计生工作突出矛盾统一起来，纳入到经济、社会发展全局之中，得到了基层党委政府的认同和支持，受到了广大计生干部和育龄群众的欢迎，给新时期人口和计生工作带来了生机和活力。
　　“三结合”的具体内容是：人口和计划生育工作同发展城乡经济相结合，同构建和谐社会相结合，同建设文明幸福家庭相结合。
　　“五关爱”的具体内容是：
　　关爱计划生育家庭，强化政策导向，稳定低生育水平。落实农村部分计划生育家庭奖励扶助制度和城镇独生子女父母退休后奖励制度，制定对独生子女家庭及计划生育家庭的优先、优惠政策。依法落实独生子女及其父母奖励和福利待遇，在独生子女入托、入学、就医、就业等方面给予优先、优惠。积极发展社会保障事业，逐步建立覆盖城乡的生育保险和养老保障制度。
　　关爱新生儿，减少出生缺陷，提高出生人口素质。全面开展优生工程，大力宣传普及优生优育知识，为群众提供优生咨询及指导服务；做好预防出生缺陷的监测筛查，实行药物干预，努力降低出生缺陷发生率。做好预防艾滋病知识的宣传，开展高危人群的艾滋病检测工作，阻断艾滋病母婴垂直传播途径。
　　关爱女孩，倡导男女平等，促进出生人口性别比平衡。深入开展“婚育新风进万家”活动、“关爱女孩行动”，在全社会营造生男生女都一样、男女平等的婚育新风。要严厉打击贩卖、残害、遗弃女婴的违法犯罪活动，坚决禁止非医学需要的胎儿性别鉴定和选择性别的人工终止妊娠的行为。采取减免学杂费等措施，帮助农村计划生育困难家庭女孩完成义务教育，为女孩成长营造良好的社会环境。
　　关爱育龄群众，增进生殖健康，建设文明幸福家庭。加强计划生育技术服务网络建设，改善服务条件，规范服务标准，增强服务能力。实施以避孕节育措施知情选择、出生缺陷干预、生殖道感染干预“三大工程”为重点的生殖健康推进计划，开展男性生殖保健服务。开发、推广避孕节育、优生优育、生殖保健的新技术和新产品，发展生殖健康产业。
　　关爱计划生育贫困人群，实施扶助创业，推进脱贫致富。在农村采取贴息贷款、项目优先、科技扶持、政策优惠等措施，帮助计划生育家庭增加收入，解决困难；在城市采取小额借款、技能培训等措施，帮助计划生育家庭解决生活困难和独生子女就业问题。组织计划生育家庭劳务输出，广开致富门路。动员城乡计生协会会员、志愿者同计划生育困难家庭开展“手拉手”、“联谊桥”等扶助活动，帮助脱贫致富。

　　二、城乡协调推进“三结合”开展“五关爱”活动的运作方式
　　我省在开展“三结合”、“五关爱”活动中探索总结了“党政领导、部门配合、项目运作、科学管理、典型引路、资金保障、全面推进”的运作方式。
　　一是坚持党政领导。2004年初，省政府办公厅转发了省人口计生委《关于城乡协调推进人口和计划生育“三结合”活动的意见》，省里成立了“五关爱”活动领导小组。全省各地都以党委、政府的名义出台活动的指导意见和活动方案。各级党政领导都把“五关爱”活动纳入全局工作，并切实承担起了在开展“五关爱”活动中的“统筹规划、政策引导、组织协调、检查督促、搞好服务”的责任。
　　二是协调部门配合。“三结合”“五关爱”活动涉及多个部门。在各级政府的统一领导下，各相关部门积极参与，互相配合，出台政策，提供资金，形成了资源共享、信息共用、责任共担、齐抓共管的局面。省人口计生委联合省发改委、财政厅、劳动保障厅、教育厅、人口计生委等8个部门下发了开展“三结合”“五关爱”活动的通知。动员省红十字会、慈善总会、计划生育协会等社会团体广泛参与，促进了计划生育行政管理、技术服务和群众工作“三位一体”互联、互补、互动的现代社会管理服务体系的建设。
　　三是实行项目运作。省里根据活动内容制定了优生工程、阻断母婴垂直传播艾滋病、计生贫困家庭女孩助学、生殖健康筛查、城镇独生子女就业培训和扶持农村计生贫困家庭致富等6个重点项目的文本。各市、州，县（市、区）及试点乡（镇）也都制定了项目文本，明确了项目的主题、任务、目标、实施步骤与具体措施、检查与评估标准、项目的负责部门和合作单位，保证活动科学有序开展。
　　四是突出科学管理。各级政府及计生部门加强了对这一活动的科学管理，建立和完善工作责任。省人口计生委把“五关爱”活动纳入了“双线”考核内容，保证了活动取得实效。松原市政府成立了由计生、卫生等部门组成的联席会议制度，对每一个项目，每个阶段的运行情况都组织专家组，按照项目文本进行科学评估。
　　五是运用典型引路。省人口计生委对“五关爱”的每一个项目，都采取先试点，后推广的办法。选取出生人口性别比较高的10个县（市）作为“关爱女孩”项目的试点单位，选取出生缺陷发生率较高的12个县（市、区）开展优生示范工程，都取得了成功的经验，推动了面上的工作。
　　六是落实资金保障。2004年以来，省政府投入了近两千万元，用于“三结合”“五关爱”项目。省红十字会、慈善总会出资50万元，用于阻断母婴垂直传播艾滋病项目；省劳动保障厅从再就业培训资金中投入200万元，用于培训失业和待业独生子女就业项目；省农村信用联社投放8738.4万元小额贷款，从主管省长基金和县（市、区）事业费中支出250万元，作为贷款贴息，用于扶助计划生育贫困人群项目；此外，各地还从社会募捐近千万元。到2005年7月底，全省用于“三结合”“五关爱”活动的资金已超过1.2亿元。

　　三、城乡协调推进“三结合”开展“五关爱”活动取得的成效
　　“三结合”“五关爱”活动开展以来，取得了明显的成效。
　　一是计划生育政策导向作用效果显著。省政府出台了《关于城镇计划生育家庭独生子女父母退休后奖励实施意见》，规定对城镇退休独生子女父母一次性奖励2000元。在农村部分计划生育家庭奖励扶助制度扩大到90%的人群基础上，由省、市、区出资解决其余10%人群的奖励扶助经费，使奖励扶助制度覆盖面达到100%。2004年全省发放独生子女父母光荣证10万多个，相当于前两年的总和，符合条件而主动放弃再生育的占应生育总数的28.27%。
　　二是新生儿出生缺陷发生率明显下降。全省筹资900余万元，引进先进设备，购置干预药物，培训技术人员。2004年共对62010名育龄妇女进行了优生筛查，对92706名育龄妇女实施了药物干预。12个优生示范县（市）的出生缺陷率下降了6.2个千分点。实施“阻断母婴垂直传播艾滋病项目”，筛查重点人群10226人，发现感染者8例，并有效进行了防控。
　　三是出生性别比失调得到有效治理。全省加强了“关爱女孩行动”的宣传，促进了群众婚育观念的转变。与省教育厅共同发文，采取免交学杂费等办法，帮助11464名辍学女孩返校学习。会同公安、卫生等部门整顿了卫生医药市场，打击了拐卖、虐待女婴、非医学需要的胎儿性别鉴定和选择性别的人工终止妊娠等违法行为。2004年，我省出生人口性别比为111.03，比“五普”时下降了0.2个百分点。
　　四是育龄群众的生殖健康水平不断提高。全省各地加强了服务网络建设，形成了以省、市服务机构为指导，县服务站为重点，乡镇中心服务站为骨干，一般乡镇服务站和村服务室为基础，服务车为纽带的技术服务网络。创建省级优质服务先进县32个，国家级优质服务先进县8个。2004年，全省各地利用各级服务机构和流动服务车，共为341.1万名育龄群众免费查病，其中患病90.2万人，治疗78.1万人。普查人群中，男性占20%以上。
　　五是部分计划生育贫困人群摆脱贫困。2004年，全省共帮扶12572户农村计划生育贫困家庭，采取贴息贷款、提供致富技术、组织劳务输出等方式，使这些计划生育家庭当年户均增收400余元。各地人口计生部门与劳动就业部门配合培训城镇失业和未就业独生子女10347名，其中90％实现就业。
　　六是促进了人口计生部门作风的转变。“五关爱”活动的开展，重塑了计生队伍爱民、为民的新形象。2004年，省人口计生委在省直27个综合部门政行风评比中，获得总体评价和本级情况两个优秀率第一。2005年，吉林省新闻媒体开展“吉林人民满意的金牌形象使者”社会调查活动，吉林省人口计生委荣获了“吉林人民满意的金牌形象使者”称号。在各地组织的政行风测评中，9个市、州人口计生部门有5个在当地50多个参评部门中位列前10名。
　　城乡协调推进“三结合”开展“五关爱”活动，突出了以人为本的理念，加快了人口计生工作“两个转变”的进程，促进了人口计生工作新机制的建立和完善，推动了覆盖城乡的计划生育服务体系的建设，体现了人口计生工作与国际社会发展目标的有效对接。

© 北大法宝：（[www.pkulaw.com](https://www.pkulaw.com/)) 专业提供法律信息、法学知识和法律软件领域各类解决方案。北大法宝为您提供丰富的参考资料，正式引用法规条文时请与标准文本核对。
欢迎查看所有[产品和服务](https://www.pkulaw.com/helps/2.html)。法宝快讯：[如何快速找到您需要的检索结果？](https://www.pkulaw.com/helps/70.html)[法宝](https://www.pkulaw.com/helps/69.html)V6有何新特色？

扫描二维码阅读原文

**原文链接：**<https://www.pkulaw.com/chl/59034e77171c6160bdfb.html>
